# Predictive Factors and *ACE-2* Gene Polymorphisms in Susceptibility to Long COVID-19 Syndrome

**DOI:** 10.3390/ijms242316717

**Published:** 2023-11-24

**Authors:** David Varillas-Delgado, Carmen Jimenez-Antona, Angel Lizcano-Alvarez, Roberto Cano-de-la-Cuerda, Alberto Molero-Sanchez, Sofia Laguarta-Val

**Affiliations:** 1 Department of Exercise and Sport Science, Faculty of Health Sciences, Universidad Francisco de Vitoria, Pozuelo, 28223 Madrid, Spain; david.varillas@ufv.es; 2 Department of Physical Therapy, Occupational Therapy, Rehabilitation and Physical Medicine, Faculty of Health Sciences, Universidad Rey Juan Carlos, Alcorcon, 28922 Madrid, Spain; carmen.jimenez@urjc.es (C.J.-A.); roberto.cano@urjc.es (R.C.-d.-l.-C.); alberto.molero@urjc.es (A.M.-S.); sofia.laguarta@urjc.es (S.L.-V.); 3 Department of Nursing and Stomatology, Faculty of Health Sciences, Universidad Rey Juan Carlos, Alcorcon, 28922 Madrid, Spain

**Keywords:** COVID-19, Long COVID-19 syndrome, early symptoms, *ACE-2* gene, polymorphisms

## Abstract

Long COVID-19 syndrome is present in 5–10% of patients infected with SARS-CoV-2, and there is still little information on the predisposing factors that lead to its development. The purpose of the study was to evaluate the predictive factors in early symptoms, clinical features and the role of Angiotensin-Converting Enzyme-2 (*ACE-2*) c.513-1451G>A (rs2106806) and c.15643279T>C (rs6629110) polymorphisms in the susceptibility to developing Long COVID-19 syndrome subsequent to COVID-19 infectionA total of 29 patients who suffered COVID-19 were recruited in a descriptive longitudinal study of two groups: Long COVID-19 (n = 16) and non-Long COVID-19 (n = 13). Early symptoms and clinical features during COVID-19 were classified by a medical service. *ACE-2* polymorphisms were genotyped by using a Single Nucleotide Primer Extension (SNPE). Of the early symptoms, fatigue, myalgia and headache showed a high risk of increasing Long COVID-19 susceptibility. Clinical features such as emergency care, SARS-CoV-2 reinfection, previous diseases, respiratory disease and brain fog also had a high risk of increasing Long COVID-19 susceptibility. The A allele in the rs2106806 variant was associated with an odds ratio (OR) of 4.214 (95% CI 2.521–8.853; *p* < 0.001), and the T allele in the rs6629110 variant was associated with an OR of 3.754 (95% CI 1.785–6.105; *p* = 0.002) of increasing Long COVID-19 susceptibility. This study shows the risk of *ACE-2* polymorphisms, different early symptoms and clinical features during SARS-CoV-2 infection in susceptibility to Long COVID-19.

## 1. Introduction

Long COVID-19 (or post-COVID-19 syndrome or long-haul COVID-19) is of recent interest in the scientific and medical communities due to the little knowledge that still exists on this disease [1]. It affects COVID-19 survivors who have overcome all stages of severity of Severe Acute Respiratory Syndrome Coronavirus 2 (SARS-CoV-2) disease and occurs approximately in 10% of SARS-CoV-2 infections [2]. Different descriptions of Long COVID-19 have already been proposed, and the most common description is symptoms lasting for more than three months after the onset of the first symptom [3]. Of the more than 200 clinical symptoms that have been described in Long COVID-19, the most common are fatigue and dyspnea (i.e., shortness of breath) [4,5], and most of them affect multiple organ systems (i.e., pulmonary and cardiovascular systems) [5,6,7,8]. The main clinical symptoms of Long COVID-19 syndrome include thrombotic events, brain fog, myocarditis, respiratory distress, fatigue, muscle aches and pains and others. Due to the binding of the virus to angiotensin-converting enzyme 2 (ACE-2) receptors expressed in many organs, it can potentially affect any system; however, it most often affects the cardiovascular, central nervous, respiratory and immune systems. Also, age, high body mass index, female sex, previous hospitalization and smoking have been shown previously to be some of its risk factors [9].

Several studies have found that Long COVID-19 occurs in even mild to moderate cases and in young adults who did not require respiratory support or hospital or intensive care [10]. Additionally, patients who tested negative for SARS-CoV-2 and were discharged from the hospital and free of disease, as well as outpatients, may also develop Long COVID-19 [11].

Early symptoms (first week of symptoms of COVID-19) such as fatigue, headache, dyspnea, hoarse voice and myalgia, among others, have been shown as the most predictive factors for developing Long COVID-19 syndrome [12]. Also, previous studies suggest that there are ethnic aspects that cause this susceptibility to SARS-CoV-2; lower infection rates are shown in Asia and Africa compared to Europe and America [13,14]. The inflammatory process due to the cytokine cascade has also been of interest in the susceptibility to SARS-CoV-2 infection, as the production of Th2, Th1/Th17 cells are key in the severity of COVID-19 [15], as with other respiratory tract diseases [16]. Genetic gateways also can determine an individual’s susceptibility to COVID-19 infection, severity, and mortality [17,18]. However, these factors in Long COVID-19 are largely unknown.

Among the most studied genetic factors in susceptibility to COVID-19 due to SARS-CoV-2 binding is the *ACE-2* gene for facilitating the entry into host cells [19,20]. The *ACE-2* gene has been shown to be a genetic risk factor for SARS-CoV-2 infection. In particular, different variations in expression show the increased susceptibility and severity of the disease [21]. Several single-nucleotide polymorphisms of the *ACE-2* gene have been observed to be associated with hypertension and other cardiovascular diseases [22] and could influence susceptibility to SARS-CoV-2 infection, especially c.513-1451G>A (rs2106806) and c.15643279T>C (rs6629110) polymorphisms [23] and G>A changes at nucleotide +4 of intron 3 (rs2285666), showing that GG and TT genotypes are significantly more susceptible to severe outcomes of COVID-19 [24]. Several association studies investigating the influence of human gene variants on COVID-19 susceptibility and severity have recently been presented to identify the mutations involved. However, the findings remain inconsistent in these gene–susceptibility association studies [25], and the molecular causes involved in the disease susceptibility are still unknown [26], showing recently that several polymorphisms in *ACE-2* do not predispose individuals to Long COVID-19 syndrome [27].

Therefore, the aim of this study was to evaluate the predictive factors in early symptoms, clinical features and the role of *ACE-2* c.513-1451G>A (rs2106806) and c.15643279T>C (rs6629110) polymorphisms in the susceptibility to developing Long COVID-19 syndrome after suffering a COVID-19 infection. We hypothesize that the *ACE-2* gene polymorphisms previously presented may be related to Long COVID-19, as has been shown with COVID-19 susceptibility. Also, early symptoms and clinical features of COVID-19 could play a relevant role in the development of this syndrome.

## 2. Results

The statistical power was 80%, with the type I error set at 5%. The *ACE-2* polymorphisms analyzed met the HWE (all *p* > 0.050) (Table 1).

### 2.1. Early Symptoms

Table 2 depicts the frequencies of early symptoms of COVID-19 between the two groups, and differences were observed between the two groups in terms of fatigue (*p* = 0.004), myalgia (*p* = 0.006) and headache (*p* = 0.013). Binary logistic regression analysis showed an association that fatigue had an odds ratio (OR) of 2.708 (95% Confidence Interval (CI): 1.263–5.086; *p* = 0.008), myalgia an OR of 2.444 (95% CI: 1.479–4.039; *p* = 0.001) and headache an OR of 2.300 (95% CI: 1.443–3.665; *p* = 0.003) of increasing Long COVID-19 susceptibility.

### 2.2. Clinical Features

Table 3 depicts the frequencies of clinical features after COVID-19 diagnosis in both groups. Differences were observed between groups in terms of time of symptoms (*p* < 0.001), emergency care (*p* < 0.001), SARS-CoV-2 reinfection (*p* = 0.013), previous diseases (*p* = 0.003), respiratory disease (*p* = 0.013) and brain fog (*p* = 0.003). Binary logistic regression analysis showed an association that emergency care had an OR of 9.705 (95% CI: 1.452–16.086; *p* = 0.003), SARS-CoV-2 reinfection an OR of 2.211 (95% CI: 1.343–3.265; *p* = 0.014), previous diseases an OR of 2.625 (95% CI 1.522–4.528; *p* < 0.001), respiratory disease an OR of 2.412 (95% CI: 1.313–4.421; *p* = 0.008) and brain fog an OR of 2.842 (95% CI 1.627–4.885; *p* < 0.001) for increased Long COVID-19 susceptibility.

Only one patient required hospitalization, and fourteen (48.3%) had a mild infection.

Table 4 depicts no associations with clinical features or *ACE-2* genotypes for both polymorphisms in patients who had Long COVID-19 syndrome.

### 2.3. ACE-2 Susceptibility

In *ACE-2* polymorphisms, frequencies between the groups show differences in both polymorphisms analyzed. In the dominant model, the A allele in the rs2106806 variant (AA or GA vs. GG genotype) was associated with an increased risk of being susceptible to Long COVID-19 with an OR of 3.188 (95% CI 1.274–7.975; *p* = 0.001). Similarly, the T allele in the rs6629110 variant (TT or TC vs. CC genotype) was associated with an of OR of 2.633 (95% CI 1.638–4.598; *p* < 0.001) for increased risk of Long COVID-19 susceptibility. In the recessive model, the T allele in the rs662910 variant (CC or TC vs. TT genotype) was associated with an OR of 2.242 (95% CI 1.367–3.571; *p* < 0.001) for increased risk of Long COVID-19 susceptibility (Table 5).

Finally, the A allele in the rs2106806 variant was associated with an OR of 4.213 (95% CI 2.521–8.853; *p* < 0.001) and the T allele in the rs6629110 variant was associated with an OR of 3.754 (95% CI 1.785–6.105; *p* = 0.002) for increased Long COVID-19 susceptibility.

## 3. Discussion

Although the influence of several predictor factors on susceptibility to COVID-19 have been analyzed in several previous investigations, to the authors’ knowledge, this is the first to investigate the association between genetic polymorphisms in Long COVID-19 development. The purpose of this study was to evaluate the predictive factors in early symptoms, clinical features and the role of *ACE-2* c.513-1451G>A (rs2106806) and c.15643279T>C (rs6629110) polymorphisms in the susceptibility to developing Long COVID-19 after suffering COVID-19 infection.

The main outcomes of this observational study indicate that Long COVID-19 susceptibility was associated with various early symptoms and clinical features when infected with SARS-CoV-2. These outcomes were associated with a 2- to 3-fold greater risk of Long COVID-19. Also, *ACE-2* polymorphisms (rs6629110 and rs2106806) were associated with close to a 3-fold greater risk of Long COVID-19. These results suggest that susceptibility to Long COVID-19 could be conditioned by both etiological and genetic factors.

COVID-19, caused by the SARS-CoV-2 virus, has affected millions of people worldwide. While many patients recover completely within a few weeks, a significant percentage experience persistent symptoms long after acute infection, which has been termed Long COVID-19 (post-COVID-19 syndrome or long-haul COVID-19). Long COVID-19 is a term used to describe the presence of various symptoms even weeks or months after acquiring SARS-CoV-2 infection, irrespective of the viral status [29].

Several studies have investigated the relationship between patients’ pre-existing characteristics and their risk of developing Long COVID-19. Factors such as older age [30], female gender [31] and the presence of comorbidities such as obesity, diabetes and cardiovascular disease have been repeatedly identified as possible risk factors [32].

Identifying the predictors of susceptibility to Long COVID-19 is essential to better understand the medical conditions that may lead to a compromised immune system and, therefore, a greater susceptibility to prolonged recovery and effective clinical management [33]. In this investigation, the authors explored the factors that the scientific literature has suggested are possible predictors of Long COVID-19, like fatigue, cough, chest tightness, dyspnea, palpitations, myalgia, difficulty focusing, exercise intolerance, walking intolerance, muscle pain and shortness of breath, all of which have been previously reported in Long COVID-19 syndrome [31,34,35]. In this case, respiratory problems at the onset of illness presented an association with Long COVID-19 syndrome [31]. However, a shorter length of hospital stay was inversely associated with Long COVID-19 syndrome [31], as only one patient in our study required hospital admission, whereas the duration of COVID-19 symptoms was longer in patients with Long COVID-19 syndrome (Table 3).

Other symptoms such as fatigue, myalgia and neurological-like headache and brain fog are early symptoms of SARS-CoV-2 reinfection, and the previous diseases and respiratory diseases reported in this study are similar to data from a systematic review of Long COVID-19 reported by Raveendran et al. [34]. Previously, it has been presented that brain fog has significant associations with gender (female), respiratory symptoms at onset and the severity of the illness (Intensive Care Unit (ICU) admission) [36,37], which is similar to data reported by the authors in the Spanish population.

The severity of acute COVID-19 infection has also been proposed as an important predictor of Long COVID-19 syndrome at least 6 months after discharge [38]. Patients who experience more severe disease during the acute phase appear to have a higher risk of developing persistent symptoms [39,40]. Previous studies have shown that those who require hospitalization, mechanical ventilation or other intensive medical interventions are more likely to experience prolonged symptoms. This may be related to the more extensive damage to organs and systems during the acute phase, which can leave long-term sequelae [41,42,43,44].

Individual immune response has also been investigated as a possible predictor. Some studies suggest that abnormal or dysregulated immune responses may be related to the persistence of symptoms, showing that cytokine profiles indicated slightly elevated pro-inflammatory cytokine levels in recovered patients in contrast to patients with Long COVID-19 [45,46].

Several polymorphisms in the *ACE-1* and *ACE-2* genes that may be associated with susceptibility to COVID-19, and even its severity, have been previously presented [47] as well as the prevalence of these markers and their global variations in this disease, although, to date, no definitive results have been presented and more research is needed to deepen this knowledge [24,48]. However, to date, no research in this area shows evidence to fully understand these relationships, and this study presents, for the first time, several polymorphisms in the *ACE-2* gene involved previously in COVID-19 susceptibility [23]. The *ACE-2* gene acts as a receptor on the surface of cells, and it is through this interaction that the SARS-CoV-2 virus enters in human cells [20]. The relationship between COVID-19 susceptibility and severity has been of great interest [49,50,51], and previous studies have suggested that higher levels of *ACE-2* expression may be associated with greater ease of viral entry and, thus, greater severity of COVID-19, and this may be the result of a complex interaction between multiple factors, including the host immune response [52,53].

In addition, certain genetic variations may predispose an individual to an increased susceptibility to Long COVID-19. In this sense, the *ACE-2* rs2106806 polymorphism has been shown as a transcription regulatory network within the *ACE-2* locus in the context of SARS-CoV-2 infection, finding a correlation in the Spanish population between non-infected and highly exposed healthcare workers [23]; still, no correlation was found with Long COVID-19 syndrome. This study shows that the A allele within the c.513-1451G>A rs2106806 polymorphism increases the risk of Long COVID-19 susceptibility in COVID-19 patients three-fold, as previously shown [23]. The c.15643279T>C rs6629110 polymorphism also presented an association between susceptibility and SARS-CoV-2 infection [23], with this being the first report linking the T allele of the rs6629110 polymorphism to two-fold increases in the risk of Long COVID-19 susceptibility. These findings of *ACE-2* polymorphisms need to be confirmed for external validity in future studies in larger cohorts of patients. In recent years, new research has presented genetic markers implicated in the susceptibility to and severity of COVID-19 [24,54,55,56] and in the susceptibility and severity of hospitalized patients with syndrome Long COVID-19, but there was no association with different *ACE-2* and transmembrane protease serine-2 (*TMPRSS2*) receptor polymorphisms [27]. These findings of new polymorphisms should be considered in the future to expand the knowledge of the association of genetic markers in the development of Long COVID-19 syndrome and to evaluate the role of several genetic variants in susceptibility to SARS-CoV-2 infection that could help identify more vulnerable individuals for Long COVID-19 syndrome.

The current scientific literature supports the notion that several factors interact to determine an individual’s susceptibility to Long COVID-19 syndrome. Age, gender, comorbidities, severity of acute infection, immune and genetic responses, as well as psychological and social aspects, may all play a role in the evolution of this condition. However, it is important to note that research on Long COVID-19 syndrome is constantly evolving and there are still gaps in our understanding. Future studies should address these complexities to provide a more complete picture of the predictors and ultimately improve the prevention and management of this syndrome.

This investigation presents several limitations: (i) the sample size is small due to the scarcity of Long COVID-19 cases in the first COVID-19 wave, with the established criteria and the accessibility to patients making it difficult to recruit study patients; (ii) the findings in predictors and genetic markers of Long COVID-19 syndrome must be validated in larger studies; and (iii) the genetic markers are an approximation of the current state, and association studies with new markers will be conducted in the future to complete our understanding of how host genetics may condition the risk of Long COVID-19 syndrome.

## 4. Materials and Methods

### 4.1. Study Design

A descriptive longitudinal study was conducted to analyze early symptoms, clinical features after suffering COVID-19 and genotype frequencies of *ACE-2* polymorphisms in Long COVID-19 and non-Long COVID-19 groups.

### 4.2. Patients

During February and March 2022, 29 patients were recruited and divided into a Long COVID-19 group (n = 16) and non-Long COVID-19 group (n = 13). The characteristics of patients in both groups are shown in Table 6.

The following inclusion criteria were established: (i) patients between 30 and 60 years of age who have previously experienced COVID-19 disease, (ii) complete COVID-19 vaccination schedule and (iii) patients provided data from previous medical history related to the processes of time of evolution, syndrome characteristics and treatments performed. The exclusion criteria were as follows: (i) the presence of chronic fatigue, (ii) the presence of uncontrolled cardiac, hypertension or respiratory diseases, (iii) the presence of disabling neurological diseases and (iv) a diagnosis or symptoms of dysautonomia. The sample size of the group of subjects with Long COVID-19 was limited since, in Spain, on the dates of recruitment of these patients, there were not yet enough with a diagnosis and clinical characteristics in accordance with the criteria for inclusion in the study due to the fact that all the subjects belonged to the first wave of COVID-19.

All patients agreed to participate in the study and consented to publishing by giving written informed consent. The protocol of the study was approved by the Ethics Committee of Hospital Universitario Fundación Alcorcón (Ethical Approval Code 21/175), and the confidentiality of the participants was guaranteed, in compliance with the Declaration of Helsinki 1964 (last updated 2013).

### 4.3. DNA Sample and Genotyping

The samples were collected with SARSTED swabs via buccal smear and kept refrigerated until genotyping.

DNA extraction from the swabs was carried out via automatic extraction using QIACube equipment (QIAGEN, Venlo, The Netherlands), yielding a DNA concentration of 25–40 ng/mL, which was kept in a solution in a volume of 100 μL at −20 °C until genotyping.

*ACE-2* c.513-1451G>A (rs2106806) and c.15643279T>C (rs6629110) polymorphisms were genotyped by using a Single Nucleotide Primer Extension (SNPE) with the SNaPshot Multiplex Kit (Thermo Fisher Scientific, Waltham, MA, USA) and analysis of the reaction result using capillary electrophoresis fragments in an ABI3500 unit (Applied Biosystems, Foster City, CA, USA), with bioinformatic analysis performed using GeneMapper 5.0 software (Applied Biosystems, Foster City, CA, USA).

### 4.4. Clinical Data

All patients were confirmed for SARS-CoV-2 infection using polymerase chain reaction (PCR) in nasopharyngeal swabs within the first 7 days from the onset of symptoms.

The medical service collected the clinical features of the participants: time of symptoms (months), hospital admission, pneumonia, emergencies, SARS-CoV-2 reinfection, previous diseases and respiratory disease. Early symptoms (first week of symptoms) of COVID-19 were classified from the study patients as “fatigue, myalgia, headache, dyspnea, arthralgia, chest pain, tachycardia and brain fog”.

### 4.5. Statistical Analysis

Analyses were performed by using the Statistical Package for the Social Sciences (SPSS), v.25.0 for Windows (IBM Corp. Released 2012. IBM SPSS Statistics for Windows, Version 25.0., Armonk, NY, USA). The disequilibria of both polymorphisms were estimated by using the Hardy–Weinberg Equilibrium (HWE) followed by the 1000 Genomes Project approach. The frequencies of clinical characteristics were compared between both groups using a chi-square test. To determine what genotype of *ACE-2* was associated with an unexpected distribution, the standardized residuals were calculated based on the difference between the observed and the expected values. Briefly, within each model, a genotype was considered to have a statistically different distribution from the expected value when its distribution was > or < the critical Z-score value (i.e., 1.96). The significance level in the univariate analysis was set at *p* < 0.050. For each *ACE-2* polymorphism, a logistic regression analysis was calculated with 95% CI to perform the assessment of the genetic association with susceptibility. For this purpose, both polymorphisms were coded under a dominant and recessive genetic model in which both heterozygous and rare allele homozygous were combined and compared without correction for multiple comparisons with the genotype homozygous in both cases [23]. To reduce the likelihood of type I error, we applied a Bonferroni correction with a significance level of *p* < 0.004.

## 5. Conclusions

In this study, the authors present, for the first time, the association between the *ACE-2* c.513-1451G>A rs2106806 and c.15643279T>C rs6629110 polymorphisms and the risk of susceptibility to Long COVID-19 syndrome, together with the risk of different early symptoms and clinical features of SARS-CoV-2 infection in the development of this syndrome. These findings have important clinical implications for the early diagnosis of Long COVID-19 syndrome in current SARS-CoV-2-infected patients with mild to moderate symptomatology.

## Figures and Tables

**Table 1:** Genomic location and minor allele frequency (MAF) of *ACE-2* polymorphisms in Long COVID-19.

Symbol	Gene	Polymorphism	dbSNP	Genomic Location	MAF Long COVID-19	MAF (IBS) *	HWE	FIS
*ACE-2*	Angiotensin-converting enzyme 2	c.513-1451G>A	rs2106806	Xp22.2	43.8% (G)	58.1% (G)	*p* = 0.069	0.41
*ACE-2*	Angiotensin-converting enzyme 2	c.15643279T>C	rs6629110	Xp22.2	34.4% (T)	41.9% (T)	*p* = 0.135	0.24

IBS, Iberian population in Spain * Reprinted/adapted with permission from Ref. [28]. 2020, Yates A.D.; FIS, inbreeding coefficient; HWE, Hardy–Weinberg equilibrium; MAF, minor allele frequency; SNP, single nucleotide polymorphism.

**Table 2:** Early symptoms of patients after suffering COVID-19 in both groups.

		Long COVID-19 (n = 16)	Non-Long COVID-19 (n = 13)	*p* Value
Fatigue	No, n (%)	5 (31.2)	11 (84.6)	0.004
	Yes, n (%)	11 (68.8)	2 (15.4)	
Myalgia	No, n (%)	9 (56.2)	13 (100.0)	0.006
	Yes, n (%)	7 (43.8)	0 (0.0)	
Headache	No, n (%)	10 (62.5)	13 (100.0)	0.013
	Yes, n (%)	6 (37.5)	0 (0.0)	
Dyspnoea	No, n (%)	13 (81.2)	12 (92.3)	0.390
	Yes, n (%)	3 (18.8)	1 (7.7)	
Arthralgia	No, n (%)	12 (75.0)	13 (100.0)	0.052
	Yes, n (%)	4 (25.0)	0 (0.0)	
Chest pain	No, n (%)	13 (81.2)	13 (100.0)	0.099
	Yes, n (%)	3 (18.8)	0 (0.0)	
Tachycardia	No, n (%)	13 (81.2)	13 (100.0)	0.099
	Yes, n (%)	3 (18.8)	0 (0.0)	
Brain fog	No, n (%)	8 (50.0)	13 (100.0)	0.003
	Yes, n (%)	8 (50.0)	0 (0.0)	

**Table 3:** Clinical features of patients after suffering COVID-19 in both groups.

		Long COVID-19 (n = 16)	Non-Long COVID-19 (n = 13)	*p* Value
Time of symptoms, months (SD)		19.69 (4.13)	0.61 (0.12)	<0.001
Hospital admission	No, n (%)	15 (93.8)	13 (100.0)	0.359
	Yes, n (%)	1 (6.2)	0 (0.0)	
Pneumonia	No, n (%)	13 (81.2)	13 (100.0)	0.099
	Yes, n (%)	3 (18.8)	0 (0.0)	
Emergencies	No, n (%)	4 (25.0)	12 (92.3)	<0.001
	Yes, n (%)	12 (75.0)	1 (7.7)	
SARS-CoV-2 reinfection	No, n (%)	10 (62.5)	13 (100.0)	0.013
	Yes, n (%)	6 (37.5)	0 (0.0)	
Previous diseases	No, n (%)	8 (50.0)	12 (92.3)	0.003
	Yes, n (%)	8 (50.0)	1 (7.7)	
Respiratory disease	No, n (%)	9 (56.2)	13 (100.0)	0.006
	Yes, n (%)	7 (43.8)	0 (0.0)	

SD, standard deviation.

**Table 4:** Genotype association for clinical features in patients with Long COVID-19 syndrome.

		*ACE-2* c.513-1451G>A (rs2106806)				*ACE-2* c.15643279T>C (rs6629110)			
		GG	GA	AA	*p* Value	CC	CT	TT	*p* Value
Hospital admission	Yes, n (%)	0 (0.0)	0 (0.0)	1 (20.0)	0.309	1 (50.0)	1 (14.3)	2 (28.6)	0.315
	No, n (%)	3 (100.0)	8 (100.0)	4 (80.0)		1 (50.0)	6 (85.7)	5 (71.4)	
Pneumonia	Yes, n (%)	0 (0.0)	2 (25.0)	1 (20.0)	0.637	1 (50.0)	1 (14.3)	1 (14.3)	0.481
	No, n (%)	3 (100.0)	6 (75.0)	4 (80.0)		1 (50.0)	6 (85.7)	6 (85.7)	
Emergencies	Yes, n (%)	3 (100.0)	5 (62.5)	4 (80.0)	0.420	2 (100.0)	5 (71.4)	5 (71.4)	0.683
	No, n (%)	0 (0.0)	3 (37.5)	1 (20.0)		0 (0.0)	2 (28.6)	2 (28.6)	
SARS-CoV-2 reinfection	Yes, n (%)	0 (0.0)	3 (37.5)	3 (60.0)	0.237	0 (0.0)	4 (57.1)	2 (28.6)	0.274
	No, n (%)	3 (100.0)	5 (62.5)	2 (40.0)		2 (100.0)	3 (42.9)	5 (71.4)	
Previous diseases	Yes, n (%)	1 (33.3)	4 (50.0)	3 (60.0)	0.766	1 (50.0)	4 (57.1)	3 (42.9)	0.734
	No, n (%)	2 (66.7)	4 (50.0)	2 (40.0)		1 (50.0)	3 (42.9)	4 (57.1)	
Respiratory disease	Yes, n (%)	1 (33.3)	4 (50.0)	2 (40.0)	0.866	0 (0.0)	5 (71.4)	2 (28.6)	0.111
	No, n (%)	2 (66.7)	4 (50.0)	3 (60.0)		2 (100.0)	2 (28.6)	5 (71.4)	

**Table 5:** Association between the *ACE-2* polymorphisms and Long COVID-19 susceptibility.

		*ACE-2* c.513-1451G>A (rs2106806)												
		Genotype			Dominant Model		Recessive Model		Allele		OR [95% CI]; *p* Value			
		GG	GA	AA	GG	GA + AA	AA	GA + GG	G	A	Genotype	Dominant Model	Recessive Model	Allele
Long COVID-19	Yes, n (%)	3 (18.8) ↓	8 (50.0)	5 (31.2) ↑	3 (18.8) ↓	13 (81.2) ↑	5 (31.2)	11 (68.8)	3 (18.7) ↓	13 (81.3) ↑	<0.001	3.188 [1.274–5.975]; 0.001	1.574 [0.952–2.631]; 0.074	4.214 [2.521–8.853]; <0.001
	No, n (%)	9 (69.2) ↑	3 (23.1)	1 (7.7) ↓	9 (69.2) ↑	4 (30.8) ↓	1 (7.7)	12 (92.3)	9 (69.2) ↑	4 (30.3) ↓				
		***ACE-2* c.15643279T>C (rs6629110**)												
		**Genotype**			**Dominant Model**		**Recessive Model**		**Allele**		**OR [95% CI]; *p* value**			
		**CC**	**CT**	**TT**	**CC**	**CT + TT**	**TT**	**CT + CC**	**C**	**T**	**Genotype**	**Dominant Model**	**Recessive Model**	**Allele**
Long COVID-19	Yes, n (%)	2 (12.4) ↓	7 (43.8)	7 (43.8) ↑	2 (12.5) ↓	14 (87.5) ↑	7 (43.8) ↑	9 (56.2)	2 (12.5) ↓	14 (87.5) ↑	0.001	2.633 [1.638–4.598]; <0.001	2.242 [1.367–3.571]; <0.001	3.754 [1.785–6.105]; 0.002
	No, n (%)	7 (53.8) ↑	5 (38.5)	1 (7.7) ↓	7 (53.8) ↑	6 (46.2) ↓	1 (7.7) ↓	12 (92.3)	7 (53.8) ↑	6 (46.2) ↓				

CI, confidence interval; OR, odds ratio. The *p* values found through allele, dominant and recessive models were adjusted using the Bonferroni correction method. ↑ indicates that the frequency was above the expected value, calculated from the standardized residuals of a chi-square test.; ↓ indicates that the frequency was below the expected value, calculated from the standardized residuals of a chi-square test.

**Table 6:** Patients’ characteristics in Long COVID-19 and non-Long COVID-19 groups.

		Long COVID-19 (n= 16)	Non-Long COVID-19 (n = 13)	*p* Value
Age, years (SD)		46.13 (7.91)	46.92 (6.00)	0.766
Weight, kg (SD)		65.52 (12.52)	63.14 (13.39)	0.363
Height, cm (SD)		166.23 (8.03)	167.25 (7.99)	0.743
Sex	Male, n (%)	1 (6.2)	4 (30.8)	0.082
	Female, n (%)	15 (93.8)	9 (69.2)	

cm, centimeters; kg, kilogram; SD, standard deviation.

## Data Availability

The data presented in this study are available on request from the corresponding author. The data are not publicly available due to legal restrictions.
